# Gestational Neuro-Otological Manifestation Patterns Across Trimesters in Karachi, Pakistan: A Retrospective Cross-Sectional Study

**DOI:** 10.7759/cureus.100373

**Published:** 2025-12-29

**Authors:** Shanila Feroz, Laiba Khan, Shaista B Anwar, Aouj Fatima, Marion Samson, Kalpana Parkash

**Affiliations:** 1 Otolaryngology - Head and Neck Surgery, Creek General Hospital, Karachi, PAK; 2 Obstetrics and Gynaecology, Creek General Hospital, Karachi, PAK; 3 Otolaryngology, Creek General Hospital, Karachi, PAK

**Keywords:** low- and middle-income country, neurology, neuro-otological manifestations, otology, pregnancy, public health and safety, trimester

## Abstract

Objective: To determine the prevalence and trimester-specific trends of neuro-otological manifestations among pregnant women attending antenatal care in tertiary care hospitals at Karachi, Pakistan.

Methodology: A prospective, multicenter observational study was conducted from January 2020 to December 2024 at two tertiary hospitals in Karachi. A total of 1,230 pregnant women aged 18-45 years were enrolled through non-probability consecutive sampling. Data were collected using a validated three-part assessment tool encompassing demographic characteristics, otological symptoms, and neurological/vestibular presentations. Statistical analysis was performed using SPSS software version 26 (IBM Corp., Armonk, NY). Chi-square and Fisher’s exact tests were applied, with significance defined as *P* < 0.05.

Results: Neuro-otological symptoms were highly prevalent. Otological complaints were reported in 874 (71%) participants, most commonly tinnitus in 390 (31.7%) and ear fullness in 310 (25.2% ). Neurological and vestibular manifestations were observed in 847 (69%), with vertigo (304, 24.7%), headache (260, 21.1%), and dizziness (215, 17.5%) being the most frequent. A significant increase in the frequency of symptoms was noted with advancing gestational age, with the third trimester demonstrating the highest burden across most manifestations (*P* < 0.001).

Conclusions: Neuro-otological manifestations are common during pregnancy and exhibit a trimester-dependent rise, peaking in late gestation. Routine screening and early recognition within antenatal care may help prevent misdiagnosis, reduce patient distress, and improve maternal quality of life.

## Introduction

Pregnancy represents a dynamic physiological state characterized by extensive hormonal, vascular, and metabolic modifications that support fetal growth and maternal physiological adaptation [1-2]. Increased concentrations of estrogen, progesterone, and human chorionic gonadotropin (hCG) exert widespread systemic influences, including on the central nervous system and auditory-vestibular pathways [3]. Concurrent shifts in plasma volume, vascular resistance, and microcirculatory function, particularly within the brain and inner ear, may alter neurological and sensory processing, including cochlear and vestibular function [4-5]. As a result, pregnant women frequently experience neuro-otological symptoms, which may manifest as tinnitus, vertigo, dizziness, sensorineural hearing loss, and headaches, including migraine-type episodes [6-7]. While many of these manifestations are temporary and self-limiting, some may substantially impair daily functioning or resemble more serious clinical conditions. Evidence from global literature, consisting of observational research and narrative reviews, has reported a notable prevalence of auditory and vestibular disturbances among pregnant populations [8-12]. Nevertheless, despite their relevance, neuro-otological symptoms during pregnancy remain underrecognized and insufficiently documented in maternal healthcare contexts, where they may be misinterpreted as nonspecific pregnancy-related discomforts. The existing body of research is relatively limited in scale and geographical diversity, with conspicuous data gaps in South Asian regions, particularly in resource-limited healthcare environments where routine antenatal services may not prioritize evaluation of such symptoms. This lack of epidemiological insight may delay diagnosis and management, thereby contributing to maternal distress, diagnostic uncertainty, or potentially avoidable clinical investigations. Prompt recognition of these symptoms is essential to differentiate benign pregnancy-related changes from more severe conditions, such as preeclampsia, cerebral venous thrombosis, or vestibular neuritis. Incorporating neuro-otological screening into routine antenatal care may therefore enhance diagnostic accuracy, reduce maternal anxiety, and improve quality of life outcomes. In this context, the present study seeks to assess the prevalence and clinical patterns of neuro-otological symptoms across different trimesters among pregnant women attending antenatal clinics at multiple hospitals in Karachi, Pakistan.

## Materials and methods

Study setting, design, and duration

This multicenter, prospective, longitudinal observational study was carried out over five years (January 1, 2020, to December 31, 2024) at two tertiary care hospitals in Karachi, Pakistan: Creek General Hospital (CGH) and United Hospital (UH), both affiliated with United Medical and Dental College (UMDC). The study was jointly overseen by the Departments of Otorhinolaryngology and Obstetrics and Gynecology. Ethical clearance was granted by the Institutional Review Board (IRB) of UMDC before data collection (Approval No. UMDC/Ethics255). All procedures were conducted in accordance with the principles of the Declaration of Helsinki (2013 revision), ensuring the dignity, autonomy, and safety of all participants. Written informed consent was obtained from each participant following a detailed explanation of the study objectives.

Sample size and sampling method

The minimum required sample size was determined using Cochran’s formula for estimating a single population proportion [13]:

\[ n = \frac{Z^{2} \times p \times (1 - p)}{d^{2}} \] where *n* represents the sample size, *Z* corresponds to the 95% confidence level (1.96), *p* is the estimated prevalence of neuro-otological symptoms during pregnancy (0.30, based on pilot observations), and *d* denotes the permissible margin of error (0.05). Accounting for a potential 10%-15% attrition or incomplete data, a total of 1,230 pregnant women were enrolled using a non-probability consecutive sampling strategy from antenatal outpatient clinics and obstetric inpatient units at CGH and UH.

Inclusion and exclusion criteria

Eligible participants included pregnant women aged 18-45 years, presenting in any trimester (first, second, or third) and receiving antenatal or inpatient obstetric care at either study site during the defined study period, with provision of written informed consent. Exclusion criteria included a prior history of neurological or otological disorders unrelated to pregnancy; previous neurosurgical interventions or significant head trauma; diagnosed psychiatric disorders likely to impair reliable symptom reporting; chronic ear conditions such as otosclerosis or chronic suppurative otitis media; active systemic diseases, including malignancy, autoimmune disorders, or immunodeficiency disorders; current systemic corticosteroid or immunosuppressive therapy; confirmed human immunodeficiency virus infection; incomplete documentation; or refusal to participate.

Study instrument and data collection procedures

Data collection was performed using a structured, three-component clinical evaluation tool collaboratively designed by Ear, Nose, and Throat (ENT), Neurologist, and Obstetrics specialists, and reviewed for content validity by three independent IRB-appointed reviewers. Part 1 of the Appendix included demographic and baseline clinical characteristics: age, parity, gestational age, trimester, hemoglobin level (g/dL), and comorbidities (anemia, hypertension, diabetes). Part 2 evaluated otological symptoms such as tinnitus, ear fullness/pressure, and sensorineural hearing loss (SNHL), with SNHL confirmed through tuning fork assessments and pure-tone audiometry where feasible. Part 3 involved neurologist-directed evaluation of neuro-vestibular symptoms, including vertigo, dizziness/lightheadedness, gait imbalance, benign paroxysmal positional vertigo (BPPV) diagnosed using the Dix-Hallpike maneuver, headache, seizure episodes, stroke confirmed via computed tomography scan/magnetic resonance imaging (CT/MRI), and restless legs syndrome diagnosed per International Restless Legs Syndrome Study Group (IRLSSG) criteria. All participants underwent systematic ENT and neurological examinations in outpatient or inpatient care settings.

Statistical analysis

Data were analyzed using IBM SPSS Statistics (version 26.0; IBM Corp., Armonk, NY). Descriptive statistics were used to summarize demographic and clinical variables. Continuous data (e.g., age, hemoglobin level) were reported as mean ± standard deviation (SD), while categorical variables (e.g., trimester, comorbidities, presence of symptoms) were presented as frequencies and percentages. Associations between pregnancy trimester and neuro-otological manifestations were examined using the chi-square test, with Fisher’s exact test applied where appropriate. A two-tailed *P*-value < 0.05 was considered statistically significant.

Ethical considerations

Strict confidentiality was maintained throughout the study. Personal identifiers were removed, and each participant was assigned a unique coded identifier for data entry and analysis. All digital and paper records were securely stored and accessible only to authorized study personnel. Participation was entirely voluntary, and participants were free to withdraw from the study at any point without affecting their clinical care.

Reporting standards

This study was conducted and reported in accordance with the STROBE (Strengthening the Reporting of Observational Studies in Epidemiology) guidelines to ensure methodological transparency and scientific rigor [14].

## Results

Participant demographics and clinical characteristics

A total of 1,230 pregnant women were included in the study from 2020 to 2024, recruited from CGH and UH in Karachi, with 624 (50.7%) enrolled from CGH and 606 (49.3%) from UH. The mean age of the participants was 28.7 ± 5.2 years. Most women were in their second trimester (522, 42.4%), followed by the third trimester (486, 39.5%), with parity ranging from 0 to 6 (median = 2). The mean hemoglobin level was 10.5 ± 1.3 g/dL, and anemia was noted in 628 (51.1%) of the study population. Additionally, 178 (14.5%) had hypertension, and 78 (6.3%) were diagnosed with diabetes mellitus (Table 1).

**Table 1:** Demographic and clinical characteristics of the participants (n = 1,230). CGH, Creek General Hospital, UH, United Hospital

Variable	*n* (%)/Mean ± SD
Age (years)	28.7 ± 5.2
Parity (median, range)	2 (0-6)
First trimester	222 (18.0%)
Second trimester	522 (42.4%)
Third trimester	486 (39.5%)
Hemoglobin (g/dL)	10.5 ± 1.3
Anemia	628 (51.1%)
Hypertension	178 (14.5%)
Diabetes mellitus	78 (6.3%)
CGH	624 (50.7%)
UH	606 (49.3%)

Prevalence of neuro-otological manifestations

Overall, 874 (71%) of participants reported at least one otological symptom, while 847 (69%) experienced one or more neurological manifestations. Among otological complaints, tinnitus was the most frequently observed (390, 31.7%), followed by sensations of ear fullness or pressure (310, 25.2%). Headache was the most common neurological symptom (260, 21.1%), whereas dizziness or lightheadedness occurred in 215 (17.5%). Vertigo, reflecting combined auditory-vestibular involvement, was reported in 304 (24.7%) (Table 2).

**Table 2:** Prevalence of gestational neuro-otological symptoms (n = 1,230). SNHL, sensorineural hearing loss; RLS, restless legs syndrome; BPPV, benign paroxysmal positional vertigo

Symptom	*n* (%)
Otological manifestations	
Tinnitus	390 (31.7%)
Ear fullness/pressure	310 (25.2%)
SNHL	174 (14.1%)
Neurological manifestations	
Dizziness/lightheadedness	215 (17.5%)
Unsteadiness/gait imbalance	162 (13.2%)
BPPV	89 (7.2%)
Headache	260 (21.1%)
Seizures	41 (3.3%)
Stroke	22 (1.8%)
RLS	58 (4.7%)
Overlapping neuro-otological manifestation	
Vertigo	304 (24.7%)

Trimester-wise distribution of neuro-otological symptoms

In the first trimester, tinnitus was the leading neuro-otological complaint (52, 23.4%), followed by vertigo (38, 17.1%) and headache (31, 14.0%). During the second trimester, tinnitus remained the most frequent symptom (172, 33.0%), with increased reporting of ear fullness (126, 24.1%) and headache (104, 19.9%). In the third trimester, tinnitus reached its highest frequency (166, 34.2%), accompanied by a marked rise in vertigo (145, 29.8%) and headache (125, 25.7%). Overall, several symptoms demonstrated a trimester-associated escalation, with the third trimester showing the greatest symptom burden across most neuro-otological categories (Table 3).

**Table 3:** Association of gestational neuro-otological manifestations with each trimester (n = 1,230). Note: The chi-square test was applied to assess statistical differences between trimesters. A *P*-value < 0.05 was considered statistically significant. SNHL, sensorineural hearing loss; RLS, restless legs syndrome; BPPV, benign paroxysmal positional vertigo

Symptom	First trimester	Second trimester	Third trimester	*P*-value
Neuro-otological manifestations				
Tinnitus	52 (23.4%)	172 (33.0%)	166 (34.2%)	<0.001
Ear fullness/pressure	41 (18.5%)	126 (24.1%)	143 (29.4%)	
SNHL	25 (11.3%)	78 (14.9%)	71 (14.6%)	
Vertigo	38 (17.1%)	121 (23.2%)	145 (29.8%)	
Dizziness/Lightheadedness	29 (13.1%)	88 (16.9%)	98 (20.2%)	
Unsteadiness/Gait imbalance	17 (7.7%)	65 (12.5%)	80 (16.5%)	
BPPV	11 (5.0%)	34 (6.5%)	44 (9.1%)	
Headache	31 (14.0%)	104 (19.9%)	125 (25.7%)	
Seizures	6 (2.7%)	14 (2.7%)	21 (4.3%)	
Stroke	2 (0.9%)	6 (1.1%)	14 (2.9%)	
RLS	5 (2.3%)	21 (4.0%)	32 (6.6%)	

## Discussion

Neuro-otological symptoms, including tinnitus, vertigo, dizziness, and headaches, are frequently encountered during pregnancy, yet they remain insufficiently recognized within routine obstetric evaluation. These manifestations are believed to arise from hormonally mediated alterations in inner-ear fluid dynamics and pregnancy-related cardiovascular changes affecting cerebral and vestibulocochlear circulation. However, such symptoms are often overlooked or misinterpreted as general pregnancy discomfort. The present prospective, multicenter study is the first of its kind in Pakistan to examine trimester-specific patterns of both otological and neurological complaints across gestation. Conducted at tertiary care hospitals in Karachi, the study systematically followed pregnant women throughout their pregnancies. The findings indicate a progressive rise in tinnitus and ear fullness, peaking in the third trimester, while dizziness and headache were more prominent later in gestation. Vertigo emerged as the most common overlapping neuro-otological symptom. Collectively, these results highlight the importance of improving clinical recognition, documentation, and referral algorithms, particularly in resource-limited healthcare settings.

Otological symptoms, especially tinnitus and vertigo, were notably prevalent in our sample, with a distinct increase in the third trimester, consistent with international literature. Swain et al. reported tinnitus and vertigo prevalences of 14.3% and 10.7%, respectively, among pregnant women in India [11], while Schmidt et al. documented a higher tinnitus prevalence of 33% in a Brazilian population [10]. The prevalence observed in our study falls between these reported ranges, which may reflect variations in genetic predisposition, environmental exposure, or methodological approaches. A similar temporal pattern was also observed: Schmidt et al. described early-onset vertigo progressing to gait instability in late pregnancy, while in our cohort, both tinnitus and definitive vertigo peaked during the third trimester [10]. This convergence suggests cumulative physiological influences across pregnancy, including increasing estrogen and progesterone levels, extracellular fluid shifts, and hemodynamic modifications that progressively alter vestibulocochlear homeostasis. Although a small number of participants demonstrated hearing loss, most cases in our study were sensorineural in nature. In contrast, Swain et al. largely reported conductive or mixed hearing losses attributed to middle-ear effusion [11]. The predominance of sensorineural patterns in our cohort may indicate pre-existing inner-ear vulnerability unmasked by pregnancy-related metabolic demands, or vascular changes affecting cochlear microcirculation. This distinction underscores the need for follow-up audiologic testing to differentiate transient gestational effects from underlying pathology. The role of elevated estrogen and progesterone in disturbing endolymphatic regulation and potentially inducing hydrops has been well-documented [6-7], alongside pregnancy-related increases in circulating blood volume that influence cochlear perfusion and vestibular sensitivity [10,15].

Neurological complaints, especially headache and dizziness, were also frequently reported. However, their prevalence in our cohort was slightly lower than that described by Ozdemir et al. and Alharthi et al., who observed rates of approximately 55% and 60% during pregnancy, respectively [16-17]. These differences may be attributable to cultural variations in symptom reporting, differential care-seeking behavior, or the grouping of overlapping neuro-otological symptoms within our assessment framework. Additionally, our study did not differentiate migraine from non-migraine headache phenotypes, though literature suggests that while migraines often improve due to hormonal stabilization during pregnancy, up to 8% of individuals may experience worsening or new-onset episodes [18]. Postpartum headache patterns, especially among women with prior migraine or preeclampsia, remain clinically significant and warrant future longitudinal exploration.

The high prevalence of anemia and hypertension observed in our cohort is also noteworthy. Previous studies report anemia in 71.5% of reproductive-aged women in Karachi, predominantly of the iron-deficiency type, whereas national and global estimates in pregnant women are 41.7% and 36.5%, respectively [19]. Anemia can impair cochlear oxygenation and has been associated with neuro-otological disturbances [20-23]. Although Pakistan’s pregnancy-related hypertension prevalence is lower than that reported in India and Mozambique [24], our cohort still demonstrated considerable hypertensive burden. Hypertension is known to contribute to symptoms such as tinnitus, headache, and visual changes, which may explain part of the neuro-otological profile observed [25-26].

Our findings have several clinical implications. First, neuro-otological symptoms are prevalent during pregnancy and often increase with gestational age; however, they are frequently dismissed as benign or attributed to psychological stress. Second, underrecognition can delay the diagnosis of serious conditions. Tinnitus or vertigo occurring in the setting of hypertension should prompt evaluation for hypertensive disorders. Third, persistent unilateral hearing loss or severe headaches should prompt referral to ENT or neurological specialists. Evidence indicates that most hypertension in pregnancy is gestational and amenable to surveillance and timely delivery [24]. Early detection via otological and neurological complaints may act as a sentinel for underlying systemic disease. In low-resource settings such as Pakistan, neuro-otological complaints are often overlooked during antenatal care due to a focus on obstetric complications and limited availability of ENT or neurological services. Our findings underscore the importance of incorporating basic neuro-otological assessments, such as history of tinnitus, vertigo, hearing loss, headaches, seizures, and RLS into routine antenatal visits. Given the high prevalence of anemia and gestational hypertension in Pakistan [19-23], early detection of neuro-otological symptoms could serve as an inexpensive screening tool to identify women requiring further investigation. Routine screening could also reduce maternal morbidity by prompting early management of conditions such as pre-eclampsia, stroke, or severe anemia, thereby improving maternal and fetal outcomes. Public health strategies should emphasize nutrition (iron and folate supplementation), hypertension control, and education about warning signs.

This study has several limitations. As a prospective observational study, it identifies associations rather than causal relationships between pregnancy and neuro-otological symptoms. The use of consecutive non-probability sampling may introduce selection bias and limit population representativeness. The study was confined to two tertiary-care centers in Karachi, potentially limiting generalizability to other regions. Although diagnostic confirmation was attempted through clinical assessment, comprehensive audiometry and neuroimaging were not feasible for all participants, raising the possibility of diagnostic misclassification. Additionally, reliance on self-reported symptoms may lead to recall or reporting bias. Finally, the absence of postpartum follow-up prevents the determination of symptom persistence or resolution. Future research should include multi-center cohorts with broader sociodemographic representation, standardized audiologic testing, and longitudinal postpartum evaluation to clarify the natural course of neuro-otological symptoms across pregnancy and beyond.

## Conclusions

This study highlights a considerable burden of neuro-otological manifestations among pregnant women, with symptoms such as tinnitus, vertigo, and headache demonstrating a clear trimester-dependent increase and peaking in the third trimester. Despite their clinical significance, these symptoms often receive limited attention during routine antenatal care, particularly in low-resource healthcare settings where the primary focus remains on obstetric and fetal complications. As a result, neuro-otological symptoms may be overlooked, potentially contributing to maternal distress, delayed recognition of underlying conditions, and inappropriate or fragmented care. Incorporating structured, symptom-based neuro-otological screening into standard antenatal assessments could facilitate earlier identification, timely referrals, and more effective management, an approach that is both practical and cost-efficient, especially in contexts with limited access to ENT or neurology specialists. Moreover, future longitudinal studies are needed to evaluate the persistence of these symptoms beyond pregnancy, to explore potential long-term sequelae, and to assess the clinical benefit of early intervention strategies. Addressing this gap could enhance maternal healthcare delivery and improve overall quality of life during and after pregnancy.

## Appendices

Appendix 

**Table 4:** Data collection form. CGH, Creek General Hospital; UH, United Hospital

Hospital: ☐ CGH ☐ UH Date: / /
Patient ID: Examiner Name:
A. Demographic and Clinical Details
Age (years):
Parity:
Gestational Age (weeks):
Trimester: ☐ 1st ☐ 2nd ☐ 3rd Hemoglobin (g/dL):
Hypertension: ☐ Yes ☐ No Diabetes Mellitus: ☐ Yes ☐ No Anemia: ☐ Yes ☐ No
B. Otological Symptoms (tick if present)
☐ Tinnitus
☐ Ear fullness or pressure
☐ Sensorineural hearing loss
C. Neurological Symptoms (tick if present)
☐ Vertigo
☐ Dizziness / Lightheadedness
☐ Unsteadiness / Gait imbalance
☐ Benign Paroxysmal positional vertigo (Dix-Hallpike positive)
☐ Headache
☐ Seizures
☐ Stroke
☐ Restless Legs Syndrome
D. Consent
Informed consent obtained: ☐ Yes ☐ No
